# Tumour stage, node stage, p53 gene status, and bcl-2 protein expression as predictors of tumour response to platin-fluorouracil chemotherapy in patients with squamous-cell carcinoma of the head and neck

**DOI:** 10.1038/sj.bjc.6600648

**Published:** 2002-12-02

**Authors:** P Fouret, S Temam, F Charlotte, J Lacau-St-Guily

**Affiliations:** Service d'Anatomie Pathologique, UPRES EA 3499, UFR Pitié-Salpêtrière, Paris 75013, France; Département de Chirurgie Cervico-Faciale, Unité des Marqueurs Génétiques du Cancer, Institut Gustave-Roussy, 94805 Villejuif, France; Service d'Oto-Rhino-Laryngologie et de Chirurgie de la Face et du Cou, Hôpital Tenon et UFR Saint-Antoine, 75012 Paris, France

**Keywords:** platin, fluorouracil, induction

## Abstract

The purpose of this study was to establish the relative contribution of tumour stage, node stage, p53 gene status, p53 expression, and bcl-2 protein expression to tumour response to platin-fluorouracil chemotherapy in 141 patients with squamous-cell carcinomas of the head and neck. Tumour response was measured at the primary site after three cycles of chemotherapy. Exons 2–10 and the coding part of exon 11 were sequenced on both strands. Bcl-2 or p53 expression was detected by immunohistochemistry. Predictor variables of objective response (reduction of at least 50% of tumour size) were tested in univariate and multivariate analyses. P53 mutations were found in 52 patients (37%). Tumour cells expressed p53 in 84 cases (59%) and bcl-2 in 25 cases (18%). T1 or T2 stage (adjusted odds ratio, 3.3; 95% confidence intervall 1.3–8.7; *P*=0.01), N0 node stage (adjusted odds ratio, 2.7; 95% confidence interval 1.1–6.4; *P*=0.03), p53 wild-type gene (adjusted odds ratio, 4 .0; 95% confidence interval 1.7–9.5; *P*=0.002), and bcl-2 protein expression (adjusted odds ratio, 20; 95% confidence interval 2.3–170; *P*=0.006), were positively associated with tumour response. P53 protein expression was not predictive of response. In conclusion, tumour stage, node stage, p53 gene status, and bcl-2 expression are independent predictors of tumour response to platin-fluorouracil in patients with squamous-cell carcinomas of the head and neck.

*British Journal of Cancer* (2002) **87**, 1390–1395. doi:10.1038/sj.bjc.6600648 www.bjcancer.com

© 2002 Cancer Research UK

## 

To establish factors linked to responses to radiotherapy or chemotherapy is currently a major goal of basic and translational research. It may help select patients for specific treatment and point to new targets for drugs. P53 protein is the product of one of the most frequently mutated tumour suppressor gene in cancer and also an important determinant of tumour cell sensitivity to DNA alterations including those induced by genotoxic drugs (Lowe and Lin, 2000; Lowe *et al*, 1993). P53 mutation has been linked to poor responses to treatment in a variety of tumour types (Aas *et al*, 1996; Bergh *et al*, 1995; Chresta *et al*, 1996; Ichikawa *et al*, 1997). Among squamous-cell carcinomas of the head and neck (SCCHN), tumours with wild-type p53 are twice as likely to respond to radiotherapy as tumours with p53 mutation (Koch *et al*, 1996). Esophageal carcinomas with p53 mutations are also poor responders to chemoradiotherapy (Ribeiro *et al*, 1998). In a previous study we were able to show that wild-type p53 gene status is a strong predictor of tumour response to platin-fluorouracil chemotherapy used in induction for treatment of loco-regionally advanced SCCHN (Temam *et al*, 2000). This result has been confirmed by others (Cabelguenne *et al*, 2000).

Although a lack of p53 mutation was the strongest predictor of tumour response in our work, we were aware that many cases with p53 mutations in our previous series were also responsive to chemotherapy. This suggested that response to chemotherapy may occur by ways other than p53-dependent drug-induced tumour cell apoptosis. Fundamental research has shown that apoptosis may be induced by both p53-dependent and p53-independent pathways (Clarke *et al*, 1993). The issue in such context – cell death or survival – seems to depend on the cellular levels of several pro- or anti- apoptotic molecules, many of which belong to a family of bcl-2 homologous proteins (Oltvai *et al*, 1993; Shimizu *et al*, 1999). Bcl-2 itself is an antiapoptotic protein, whose forced expression in cells generally protects against apoptosis (Tsujimoto *et al*, 1985). However, few studies at present have demonstrated that bcl-2 expression in tumour cells are linked to resistance to treatment except for lymphomas (Hermine *et al*, 1996; Schmitt *et al*, 2000). Actually, bcl-2 expression has been associated with a good response to treatment in some cancers (Fontanini *et al*, 1995; Joensuu *et al*, 1994; Pezzella *et al*, 1993). Bcl-2 expression correlated with favourable outcome in SCCHN treated by radiotherapy (Wilson *et al*, 1996) and better local regional control in patients treated by concurrent radiochemotherapy (Homma *et al*, 1999). Patients whose tumours expressed bcl-2, even with locoregionally advanced disease, were also shown to have a high likelihood of cure with aggressive combined modality therapy (Pena *et al*, 1999).

The present work determines the relative contribution of p53 gene status, bcl-2 expression, tumour stage, and node stage to the tumour response to platin-fluorouracil chemotherapy in a retrospective series of 141 consecutive cases of SCCHN.

## PATIENTS AND METHODS

### Patients

From September 1992 to December 1995, 276 cases of SCCHN (excluding nasopharyngeal tumours) were newly diagnosed in the Service d'Oto-Rhino-Laryngologie et de Chirurgie de la Face et du Cou (Hôpital Tenon, Paris, France). All 141 patients treated by induction chemotherapy during this period were consecutively included. Of these 141 cases, 107 patients presented with stage III or stage IV SCCHN and have been previously studied (Temam *et al*, 2000). The present study includes 34 additional patients with stage II SCCHN. Diagnosis, chemotherapy, response evaluation, radiotherapy, and surgery were performed at the same institution. The study was approved by the ethical committee.

### Treatment plan

For patients with stage III or stage IV tumours the treatment plan consisted of chemotherapy according to a modified Al Sarraf protocol followed by response evaluation at the primary tumour site, then local therapy which was either radiotherapy alone or salvage surgery and radiotherapy.

For patients with stage II tumours the treatment plan consisted of chemotherapy followed by surgery. All patients had three planned cycles of chemotherapy. Each cycle consisted of cisplatin (20 mg m^−2^ per day) administered as a continuous intravenous 24-h infusion, simultaneously with a continuous infusion of flu-orouracil (1000 mg m^−2^ per day) for 4 days. Chemotherapy was withdrawn in cases of tumour progression or in patients who did not respond after two cycles.

The final response was estimated from the last clinical and endoscopic evaluation, which was performed under general anaesthesia 2 weeks after the last cycle. Tumour shrinkage was evaluated as the decrease in the sum of the product of the largest perpendicular diameters of the measurable lesions at the primary tumour site. An objective response was defined by a decrease of at least 50% of initial tumour size.

### Sequencing of the p53 gene

DNA samples were obtained from the formalin-fixed paraffin-embedded biopsy specimens, which have been used for diagnosis. In cases with multiple biopsy specimens of the same tumour, one specimen was selected on the basis of more abundant tumour tissue or randomly chosen. Tumour tissue was microdissected with surgical blades under a binocular lens at a ×50 magnification, and digested in 100 μl buffer containing 200 μg ml^−1^ proteinase K overnight at 37°C.

The processing of slides, the preparation and storage of DNA samples, and the polymerase chain reactions (PCR) were performed in other rooms than those in which PCR products were manipulated.

Each exon was separately amplified except for exon 2 and 3 which were amplified together. Each primer pair included splice junctions. The PCR procedure and primers for exons 5–9 were as described elsewhere (Hsu *et al*, 1991). The same PCR conditions were used for exons 2–4, exon 10, and the coding part of exon 11, with specifically designed primer pairs as described (Temam *et al*, 2000). The column-purified PCR products were sequenced using fresh internal primers and fluorescent dye–labelled dideoxynucleotides (Dye-Terminator sequencing kit; Perkin Elmer, Norwalk, CT, USA). Fluorograms collected with a DNA sequencer (Applied Biosystems, Foster City, CA, USA) were analysed using the Sequence Navigator software (Applied Biosystems) to identify mutations by comparison with fluorograms from wild-type samples. Mutations were identified and confirmed in both strands in two independent amplification and sequencing experiments. Neutral mutations and known polymorphisms were not counted as mutations.

### Immunohistochemistry

Immunohistochemistry was performed in an automated processor (Techmate 500; Dako, Carpenteria, CA, USA) following standard procedures. The monoclonal antibody DO7 (Novocastra, Newcastle upon Tyne, UK) reacts strongly in formalin-fixed material with an antigenic determinant from the amino-terminal region of both the wild-type and mutant forms of p53 protein (Anwar *et al*, 1993). The monoclonal antibody clone 124 (Dako, Carpenteria, CA, USA) reacts with bcl-2 cytoplasmic protein (Pezzella *et al*, 1990).

To enhance the signal, deparaffinised sections were microwaved before being incubated with the primary antibody (p53, 1 : 200, overnight at 4°C; bcl-2, 1 : 50, 1 h at room temperature). Sections were incubated with a biotinylated goat antimouse immunoglobulin at a 1/600 dilution, then reacted with peroxydase-labelled streptavidin at a 1/800 dilution. The slides were developed with a solution containing diaminobenzidine tetrahydrochloride at a final concentration of 0.2 mg ml^−1^ and 0.005% H2 O2 in Tris-buffered saline, then counterstained with Harris haematoxylin.

Specimens were blindly and independently evaluated for p53 and bcl-2 staining by two observers (S Temam and P Fouret). Percentage of positive cells were recorded. Staining in less than 5% of tumour cells was scored as negative. All series for p53 immunostaining included immunoreactive breast carcinoma specimens as p53-positive external controls. Bcl-2 reactivity was controlled with adjacent lymphocytes. Negative controls included replacement of the monoclonal antibody with a mouse myeloma antibody of the same subclass.

### Statistical analysis

Significance testing for discrete variables was performed with the chi 2 test or the Fisher's exact test when appropriate.

Predictor variables with a *P* value less than 0.05 in the univariate analyses were submitted to multivariate analysis with response to chemotherapy as the dependant variable. Statistica software (StatSoft Inc., Tulsa, OK, USA) was used to develop the multivariate logistic-regression models. The relative risk to respond to chemotherapy was estimated by adjusted odds ratios with 95% confidence intervals.

All reported *P* values are two-sided. A value of 0.05 was chosen to indicate statistical significance.

## RESULTS

### P53 mutations

Forty mutations (37%) had been found in 107 cases of stage III or stage IV SCCHN. The analysis of 34 additional cases with stage II SCCHN yielded 12 mutations (35%), a prevalence which was not statistically different from the late stage series. These mutations are shown in Table 1

**Table 1:**
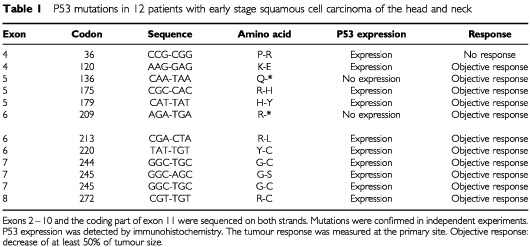
P53 mutations in 12 patients with early stage squamous cell carcinoma of the head and neck

. There was a perfect concordance between the type of mutation and p53 expression as every missense mutation expressed the protein, while the two stop mutations did not.

Among the 52 mutations, 31 (61%) were missense or inframe deletions, and the remaining 21 (39%) were predicted to interrupt the reading frame (nine stop mutations, six splice junction mutations, and six frameshift deletion or insertion mutations). A majority of mutations (83%) were localised in exons 5–9, while eight mutations were found in exon 4, and one in exon 10.

### Immunohistochemical analysis of p53 and bcl-2 protein expression **(photographic pictures available on request)**

P53 staining of tumour cells was observed in 84 patients (59%). The percentage of positive cells varied from 5–95% (median 50%).

Bcl-2 staining of tumour cells was observed in 25 patients (18%). The percentage of positive tumour cells varied from 5–90% with 12 cases having 10% or more positive cells (median 5%).

### Characteristics of patients

Most patients were male (89%) and smokers (95%). The median age was 59 years (range, 29–79 years). Fifty-six patients (40%) presented with T1 or T2 tumour stage, and 81 patients (57%) presented with N0 node stage. Tumour localisation was laryngeal in 59 cases (42%), and involved the oral cavity in seven cases (5%), the oropharynx in 52 cases (37%), and the hypopharynx in 23 cases (16%).

For the 34 patients with stage II SCCHN, tumour site was laryngeal in 19 cases, and involved the oral cavity in three cases, the oropharynx in 11 cases, and the hypopharynx in only one case.

Patients characteristics were analysed according to p53 gene status (Table 2

**Table 2:**
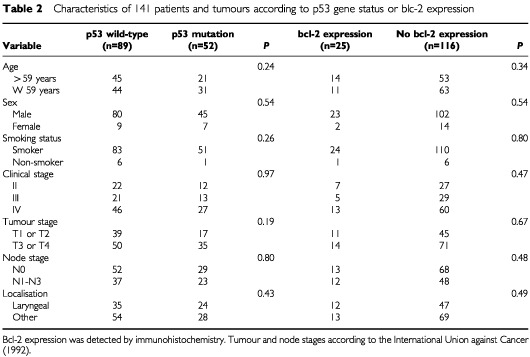
Characteristics of 141 patients and tumours according to p53 gene status or blc-2 expression

). There was no difference between patients with p53 wild-type and those with p53 mutation. The p53 mutation rate was lower for tumours of the oral cavity (one out of seven cases; 14%) and of the hypopharynx (five out of 23 cases; 22%) than for tumours of the larynx (24 out of 59 cases; 41%) and the oropharynx (22 out of 52 cases; 42%), although all differences were not significant (*P*>0.11 for all comparisons).

Similarly, there was no difference between patients whether or not they expressed bcl-2 protein (Table 2).

### Univariate analyses of tumour response

Two patients with late stage disease could not be evaluated for their tumour response and were therefore excluded from the analyses.

Among 139 patients, 99 (71%) had an objective response, and 40 (29%) did not respond. All but one patient (97%) with clinical stage II responded to chemotherapy, which was significantly higher than the proportion of responders among patients with clinical stage III or stage IV (66 out of 105, or 63%; *P*=0.0001; Table 3

**Table 3:**
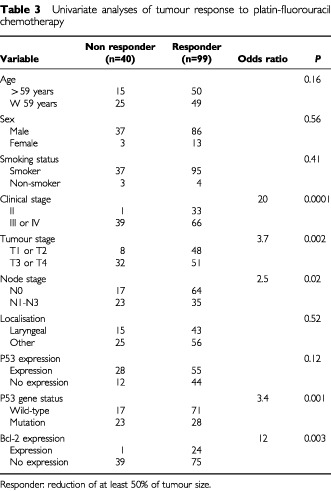
Univariate analyses of tumour response to platin-fluorouracil chemotherapy

). T1 or T2 tumour stage was significantly associated with response to chemotherapy as 48 out of 56 patients (86%) with early tumour stage were responders as compared with only 51 out of 83 patients (67%) with T3 or T4 tumour stage (*P*=0.002). Among 81 node negative patients there were 64 responders (79%), while 35 out of 58 patients (60%) with N1–N3 node stage responded to chemotherapy (*P*=0.02).

P53 protein expression was not predictive of tumour response (*P*=0.12). However, 71 out of 88 patients (81%) with wild-type p53 responded to chemotherapy in contrast to only 28 out of 51 patients (55%) with p53 mutation (*P*=0.001).

Twenty-four out of 25 patients (96%) expressing antiapoptotic bcl-2 protein (5% or more positive bcl-2 positive tumour cells) responded to chemotherapy (Table 4

**Table 4:**
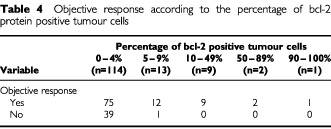
Objective response according to the percentage of bcl-2 protein positive tumour cells

). Among the remaining 114 patients whose tumour cells did not express bcl-2 protein, 75 (66%) were responders (*P*=0.001).

Twelve out of 12 patients (100%) expressing bcl-2 protein in at least 10% of tumour cells were responders. Among the remaining 127 whose tumour contained less than 10% bcl-2 positive cells, 87 (59%) were responders (*P*=0.02).

Interestingly, among 51 patients with p53 mutation 10 cases (20%) expressed bcl-2 protein. These 10 bcl-2 positive cases responded to chemotherapy despite p53 mutation. Six out of these 10 cases presented with T3 or T4 stage. In contrast among the remaining 41 cases which also had p53 mutation, but did not express bcl-2 protein, only 18 cases (44%) were responders (*P*=0.001).

### Multivariate analyses of tumour response

Univariate analyses had identified tumour stage, node stage, p53 gene status, and bcl-2 protein expression as significant predictors of tumour response. These covariates were entered in a multivariate model with tumour response as the dependant variable.

Tumour stage, node stage, p53 wild-type gene status and bcl-2 protein expression were shown to be positively associated with tumour response (Table 5

**Table 5:**
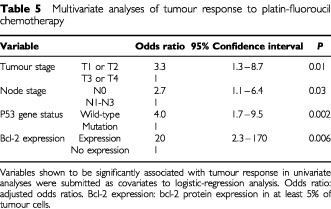
Multivariate analyses of tumour response to platin-fluoroucil chemotherapy

).

P53 wild-type gene status (adjusted odds ratio, 4.0; 95% confidence interval 1.7–9.5; *P*=0.002) was the strongest predictor of tumour response.

The correlation of bcl-2 protein expression with response was also strong, although the unique bcl-2 positive case who did not respond to chemotherapy was responsible for a very high and imprecise 95% confidence interval upper limit (95% confidence interval 2.3–170).

To test the robustness of these data, multivariate analysis was also performed using the 10% cutoff for bcl-2 protein expression. Tumour stage (adjusted odds ratio, 3.3; 95% confidence interval 1.3–8.6; *P*=0.01), node stage (adjusted odds ratio, 2.8; 95% confidence interval 1.2–6.5; *P*=0.02), p53 wild-type gene status (adjusted odds ratio, 3.9; 95% confidence interval 1.6–9.0; *P*=0.002), and bcl-2 protein expression in at least 10% of tumour cells (adjusted odds ratio, 26; 95% confidence interval 1.2–559; *P*=0.04) were also positively associated with response in this model.

## DISCUSSION

This study is an expansion of our previous study (Temam *et al*, 2000), but includes 34 new patients with earlier disease. The additional patients do correct for some deficiencies in our previous analysis of the relationships of clinical parameters as well as p53 gene status to objective response to chemotherapy. In addition, we provide immunostaining data which suggest that bcl-2 protein expression in tumour cells is a useful biological marker for predicting tumour response to induction therapy.

The 34 new patients were treated by chemotherapy and evaluated for tumour response in the same manner as patients with late disease. All coding exons of p53 gene were sequenced, revealing 12 additionnal mutations. This study indicates that the prevalence of mutations does not increase with clinical stage. Tumour and node stages were marginally predictive of tumour response in our previous study. The current study clearly indicates that tumour and node stages are clearly significant predictors of tumour response, although the correlation with response appears to be stronger for tumour stage than node stage. These clinical parameters are known predictors of tumour response in the literature. Our previous study indicated that p53 mutations are associated with poor tumour response to induction chemotherapy in patients with advanced disease. A similar conclusion has been reached by Cabelguenne *et al* (2000) in a prospective study. In the current study, p53 gene status, tumour stage, and node stage predict tumour response by multivariate analysis. Here, the contribution of clinical parameters are correctly evaluated, which strengthen the valididity of our conclusion. Strikingly, p53 gene status remains the strongest predictor of response.

The role of p53 protein in apoptosis suggests that mechanisms leading to apoptosis are important determinants of tumour response. As a first step to explore sensitivity to apoptosis in tissue samples, we assayed bcl-2 protein expression by immunostaining tumour cells with a monoclonal antibody, which is widely used and gives very consistent results in paraffin-embedded specimens (Pezzella *et al*, 1990). We did all the appropriate controls. The proportion of bcl-2 positive cases (17%) is close to those reported in other studies (Gasparini *et al*, 1995; Gallo *et al*, 1996; Wilson *et al*, 1996; Pena *et al*, 1999). The multivariate analysis shows a strong association of bcl-2 positivity (5% or more immunoreactive tumour cells) to tumour response, which is independent of tumour stage, node stage, and p53 gene status. Our scoring of bcl-2 positivity is admittedly subjective, but the predictive value of bcl-2 protein expression is also found with another cut-off (10% or more positive tumour cells). Our analysis also reveals that bcl-2 positivity predicts a good response to chemotherapy even in cases with p53 mutations. Among these cases, several presented with T3 or T4 stage, suggesting that this effect is not related to tumour stage, although we could not fit a multivariate model to the small number of observations. This suggests that bcl-2 immunoreactivity points to cases where a p53-independent response may predominate over tumour cell failure to achieve p53-dependent apoptosis.

The clinical significance of bcl-2 protein expression in SCCHN is not clearly established. Gallo *et al* (1996) reported that bcl-2 expression is closely associated with a high risk of recurrence and poor survival in stage I and II SCCHN patients. In contrast, Gasparini *et al* (1995) reported that bcl-2 SCCHN are highly responsive to concurrent chemoradiotherapy in univariate analysis. Homma *et al* (1999) also reported that in patients treated with concurrent chemoradiotherapy bcl-2 positivity predicts better locoregional control, based on a multivariate analysis. In a previous study, Wilson *et al* (1996) reported that bcl-2 expression correlates with favourable outcome in SCCHN treated by radiotherapy. The study of Pena *et al* (1999) indicated that bcl-2 positivity, even with locoregionally advanced disease, has a high likelihood of cure with aggressive combined modality therapy. Together, these studies suggest that bcl-2 protein expression in SCCHN associates with improved therapeutic response to genotoxic treatments. Our study is the first to indicate that bcl-2 positivity correlates with a better tumour response to induction chemotherapy.

Bcl-2 family proteins play pivotal roles in controlling cell death (Oltvai *et al*, 1993; Shimizu *et al*, 1999). Bcl-2 protein itself belongs to the group of prosurvival members. Therefore, it seems paradoxical that tumours expressing bcl-2 protein demonstrate an improved therapeutic response. The association of bcl-2 expression with better outcome has been reported in lung (Pezzella *et al*, 1993; Fontanini *et al*, 1995), breast (Joensuu *et al*, 1994), colon (Baretton *et al*, 1996) and ovarian carcinomas (Diebold *et al*, 1996). Expression of bcl-2 protein is common in undifferentiated carcinomas of the nasopharyngeal tract (Lu *et al*, 1993), which are nevertheless very sensitive to radiotherapy. In contrast, bcl-2 expression in lymphomas is associated with a poor response to therapy (Hermine *et al*, 1996). Bcl-2 positive lymphomas are characterised by very high levels of bcl-2 protein in 100% of tumour cells, which is related to specific translocations (Tsujimoto *et al*, 1985). It seems very likely that bcl-2 overexpression in lymphoma cells is the cause of tumour resistance to treatment (Schmitt *et al*, 2000). The context appears very different in epithelial malignancies, where bcl-2 protein expression is usually restricted to a proportion of tumour cells. In epithelial tumours the mechanisms leading to bcl-2 protein expression are certainly different from lymphomas. It is possible that bcl-2 protein expression in some epithelial malignant cells is actually a reactive change to alterations of other bcl-2 family members or other regulators of apoptosis. Fundamental research strongly suggests that tumour cell sensitivity to genotoxic damage is induced by oncogenic changes which promote apoptosis (Lowe and Lin, 2000). Bcl-2 protein expression in epithelial malignancies is probably a reactive and reversible cellular mechanism to suppress pro-apoptotic oncogenic changes, which may be stronger in some tumours.

The data from this retrospective analysis support the conclusion that bcl-2 protein expression may be a clinically relevant determinant of response of SCCHN to treatment. If dependence on bcl-2 protein is associated with greater chimiosensitivity, even in tumours with p53 mutations, the molecular alterations related to bcl-2 positivity may have important implications on the rational approach to treatment of SCCHN.
